# Endoscopic Management of Variceal Band Ligation Leading to Acute Esophageal Obstruction

**DOI:** 10.14309/crj.0000000000002111

**Published:** 2026-04-28

**Authors:** Mischelle Lopez Quinteros, Marisa S. Zayat, Jessica C. Petrov, Frank J. Lukens, Mihir S. Wagh, Jana G. Hashash, Timothy A. Woodward, Osayande Osagiede

**Affiliations:** 1 Division of Gastroenterology and Hepatology, Mayo Clinic, Jacksonville, FL

**Keywords:** esophageal varices, endoscopic variceal band ligation (EVBL), acute esophageal obstruction, dysphagia, endoscopic scissors, through-the-scope balloon dilation

## Abstract

Complete esophageal obstruction is a rare but serious complication of endoscopic variceal band ligation (EVBL). We present the case of a 75-year-old man with cirrhosis and esophageal varices who developed acute esophageal obstruction following EVBL, which was successfully managed with a combination of endoscopic scissors to cut the band and through-the-scope balloon dilation, restoring luminal patency and recanalization of the esophagus. This case highlights the importance of prevention, early recognition, and timely management of this rare but important complication of EVBL. Key management options addressed include preventive, conservative, and interventional strategies to improve patient outcomes.

## INTRODUCTION

Cirrhosis disrupts liver microcirculation, leading to increased vascular resistance within the portal system. This rise in portal pressure stimulates angiogenic factors in the splanchnic vasculature, promoting the development of portosystemic collaterals as a compensatory mechanism to decompress the portal vein. Among these, esophageal varices are the most clinically significant and feared complication. Up to 50% of patients with cirrhosis develop esophageal varices, and variceal hemorrhage carries a mortality of 15%–20%.^1–3^

Endoscopic therapy is considered the first-line treatment of this condition, with endoscopic variceal band ligation (EVBL) being one of the preferred options.^1^ EVBL is used as primary prophylaxis in patients with moderate to large varices to reduce the risk of initial bleeding, as secondary prophylaxis after an episode of variceal hemorrhage to prevent rebleeding, and in the setting of acute esophageal variceal bleeding.^4^ The procedure involves the use of a rubber band ligator with a transparent cap preloaded with bands, which is attached to the tip of the endoscope. At the target site, the variceal mucosa is suctioned into the cap where the band is released, resulting in thrombosis, necrosis, and eventually fibrotic scarring within the submucosa.^3^

Although EVBL is a minimally invasive procedure and generally safe, it is not free of complications. Reported adverse events include chest discomfort, transient dysphagia, pneumonia, postbanding ulcer bleeding, and strictures. Rarely, more severe complications, such as complete esophageal obstruction, may occur.^5^

We present an interesting case of a patient with EVBL leading to acute esophageal obstruction. The aim of our case report was to describe the diagnosis and treatment of acute esophageal obstruction, a rare but important complication of EVBL, with only around 12 cases reported in the literature to date, emphasizing the importance of early recognition and timely management.

## CASE REPORT

**Figure 1.**
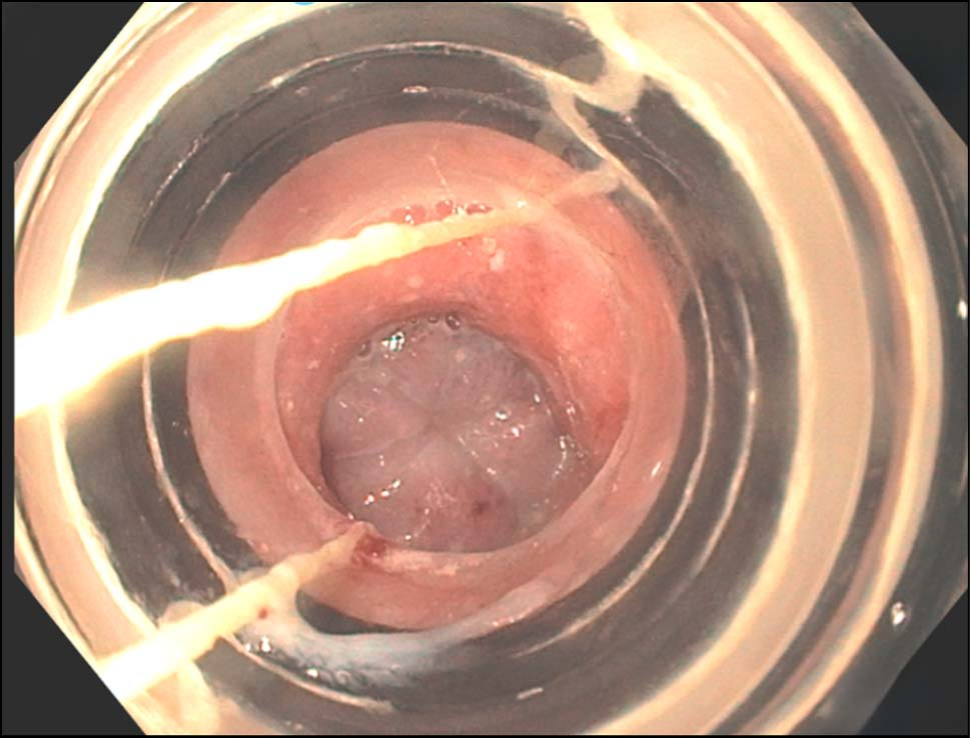
Initial esophagogastroduodenoscopy raising concern for luminal occlusion.

A 75-year-old male patient with a past medical history significant for cirrhosis secondary to chronic hepatitis C and alcohol use, portal hypertensive sequelae including esophageal varices status postbanding and portal hypertensive gastropathy, and hepatocellular carcinoma status post Y90 radioembolization presented for an elective outpatient esophagogastroduodenoscopy (EGD) for variceal surveillance. Initial EGD revealed a regular Z-line 42 cm from the incisors and 2 columns of grade II varices with no bleeding and no stigmata of recent bleeding in the distal esophagus. They were 6 mm in largest diameter. No red wale signs were present. Scarring from previous treatment was visible. Evidence of partial eradication was visible. One band was successfully placed, however, unusual button hole appearance raised concern of luminal occlusion with banding (Figure 1).

**Figure 2.**
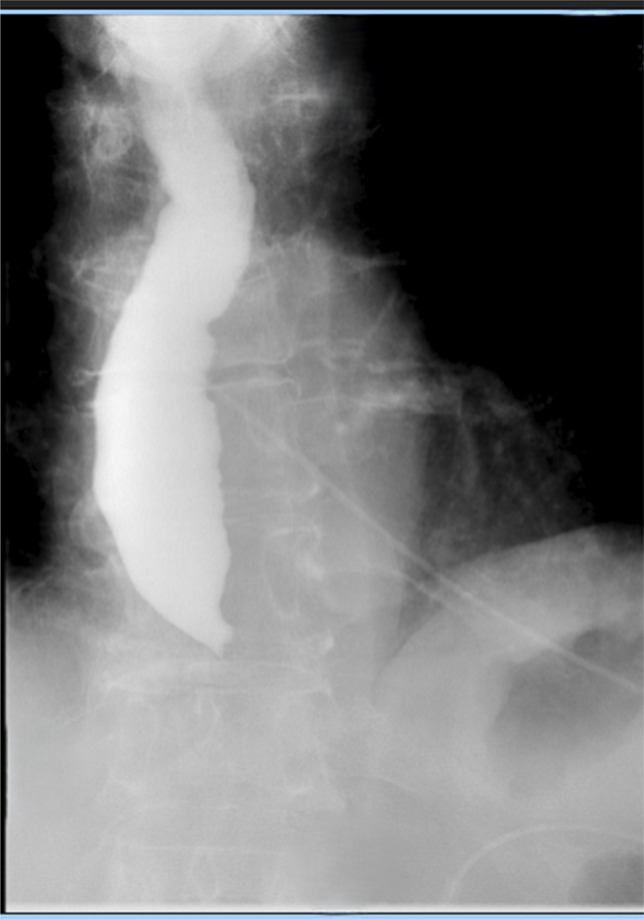
Esophagram confirming esophageal obstruction.

A single contrast barium esophagram obtained postprocedure revealed distal esophageal obstruction with mild thoracic esophageal dilation, without oropharyngeal swallowing dysfunction (Figure 2). The patient experienced dysphagia with inability to tolerate oral secretions and was admitted for monitoring.

**Figure 3.**
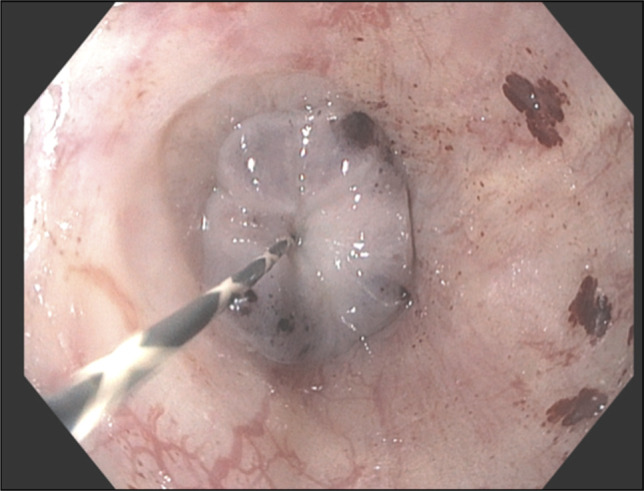
Esophagogastroduodenoscopy with a guidewire through a residual opening of only 1–2 mm.

On hospital day 1, repeat EGD demonstrated esophageal luminal stenosis related to recent circumferential capture of the esophageal wall within the banded varix, leaving a residual opening of only 1–2 mm (Figure 3). This was confirmed with a guidewire with fluoroscopy. A 16 French nasogastric tube was placed proximal to the stenosis to intermittently suction secretions.

On hospital day 2, there was no noticeable improvement in dysphagia. Patient had approximately 600 mL nasogastric tube output in suction canister. Repeat esophagram again showed no contrast passing beyond the distal thoracic esophagus.

**Figure 4.**
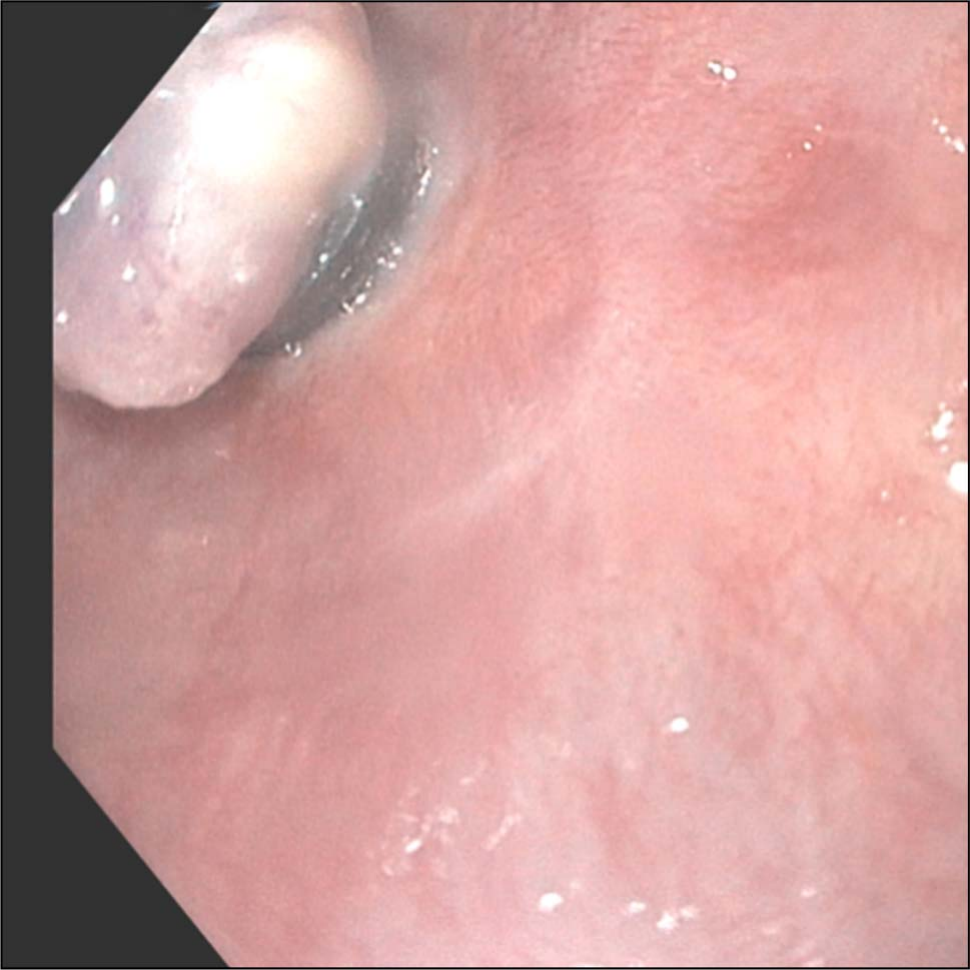
Esophagogastroduodenoscopy showing variceal band intact (side view).

**Figure 5.**
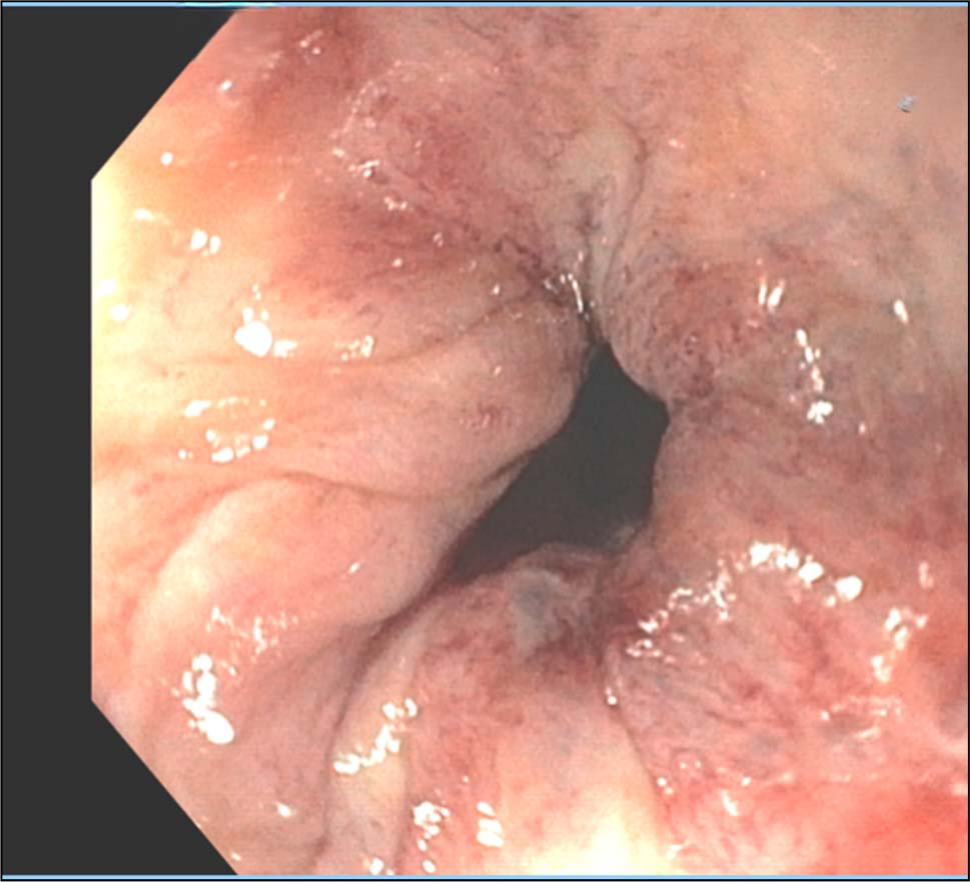
Restored luminal patency and recanalization of the esophagus.

On hospital day 3, a repeat EGD was performed. The Ensizor endoscopic scissors (Slater Endoscopy, Way Miramar, FL) were used to cut the band, restoring luminal patency and recanalization of the esophagus (Figures 4 and 5). The esophagus was then dilated using a through-the-scope balloon dilator to a maximum balloon size of 12 mm under fluoroscopic guidance. Following intervention, the patient's dysphagia resolved. The patient tolerated diet advancement and was discharged on hospital day 4.

**Figure 6.**
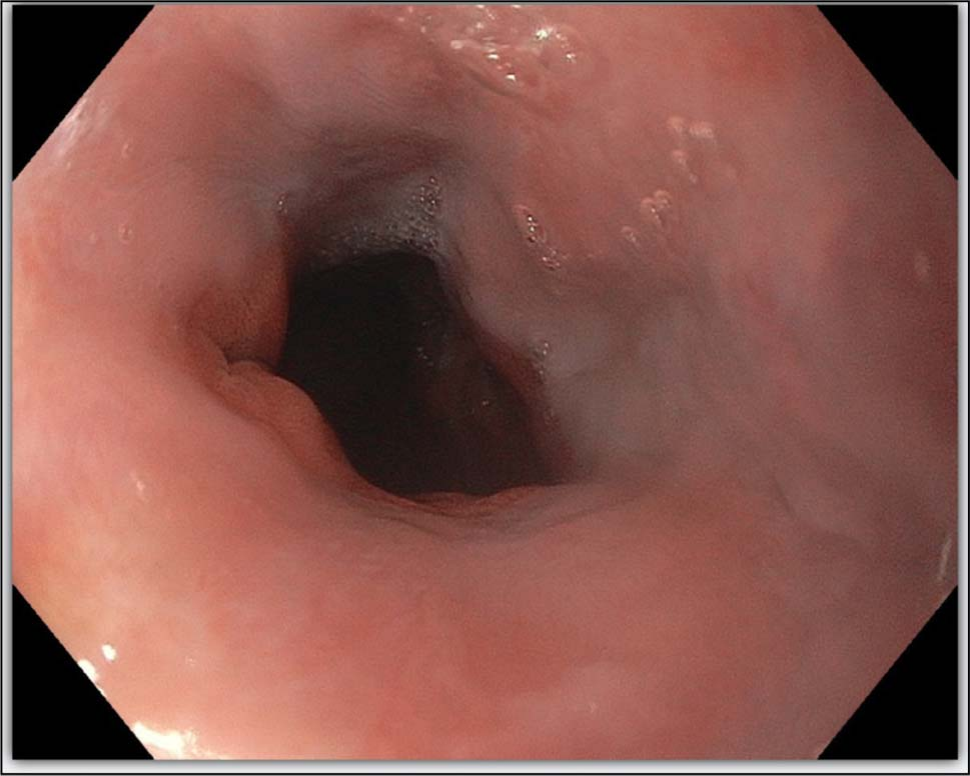
Repeat esophagogastroduodenoscopy in 6 weeks showing patent esophagus.

Repeat EGD in 6 weeks showed scarred mucosa in the lower third of the esophagus without a stricture or residual varices (Figure 6). The patient reported no dysphagia.

## DISCUSSION

Complications arising from EVBL are typically minor and self-limited, which makes the occurrence of esophageal obstruction both unexpected and clinically challenging. This rare outcome underscores the fact that even routine procedures may occasionally lead to unforeseen consequences. In this context, maintaining a high index of suspicion and promptly implementing management strategies becomes essential. Complete esophageal obstruction following EVBL has been reported in a limited number of case reports in the literature.^6–14^ Proposed mechanisms for this complication include the capture of the circumferential mucosa, edema from the banded varices, inadvertent entrapment of the opposing esophageal walls, altered motility, or the presence of residual strictures from prior banding sessions.^6,7^ In addition, positioning a band over mucosa that is already edematous, or necrotic may further predispose to luminal blockage.^8^

We speculate that the modest size of the varices with evidence of partial eradication and scarring from prior interventions may have predisposed to circumferential normal esophageal mucosal entrapment in our case. This likely resulted in excess normal tissue trapping with vigorous suctioning. Strategies that could prevent luminal obstruction and mucosal trapping in this scenario include: (i) avoiding excessive suction that captures opposing esophageal wall, circumferential mucosa, or excess normal tissue; (ii) targeting 1 variceal column at a time; and (iii) maintaining adequate insufflation before applying suction.

Management strategies described in the literature fall into 2 categories: conservative and interventional. Conservative therapy consisting of nothing by mouth, intravenous hydration, and close observation has been reported successful in several cases, with resolution typically occurring within a few days to 2 weeks.^6–11^ However, delayed recovery has been associated with late complications such as stricture formation, sometimes requiring multiple sessions of endoscopic dilation.^9^

In contrast to conservative therapy, interventional approaches aim to mechanically restore luminal patency and have included mechanical band removal, release with endoscopic scissors and esophageal stent placement, or more recently, endoscopic mucosal resection.^7,12–14^ While these interventions often achieved rapid symptom relief and diet advancement, isolated reports have described adverse events such as bleeding, dissection, or stricture formation.^7,12^ The optimal timing of intervention remains uncertain, with some authors advocating for early endoscopic management to shorten hospitalization, while others emphasize the safety of observation in stable patients. Although removing the band on the index endoscopy was considered in our case, it was ultimately decided not safe because of the risk of severe life-threatening variceal bleeding with premature band dislodgement.

**Figure 7.**
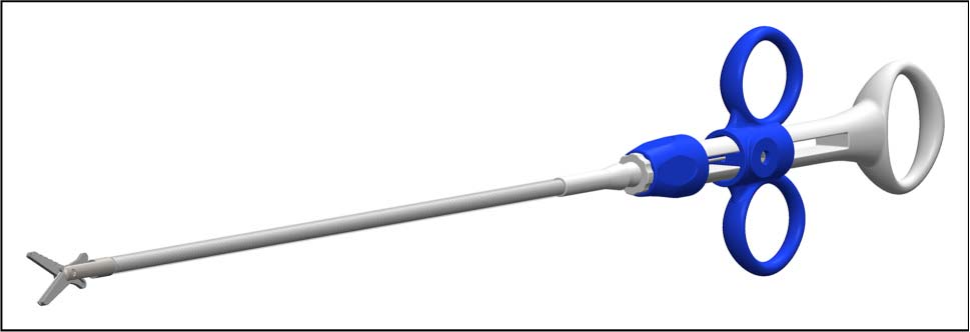
The Ensizor endoscopic scissors.

**Figure 8.**
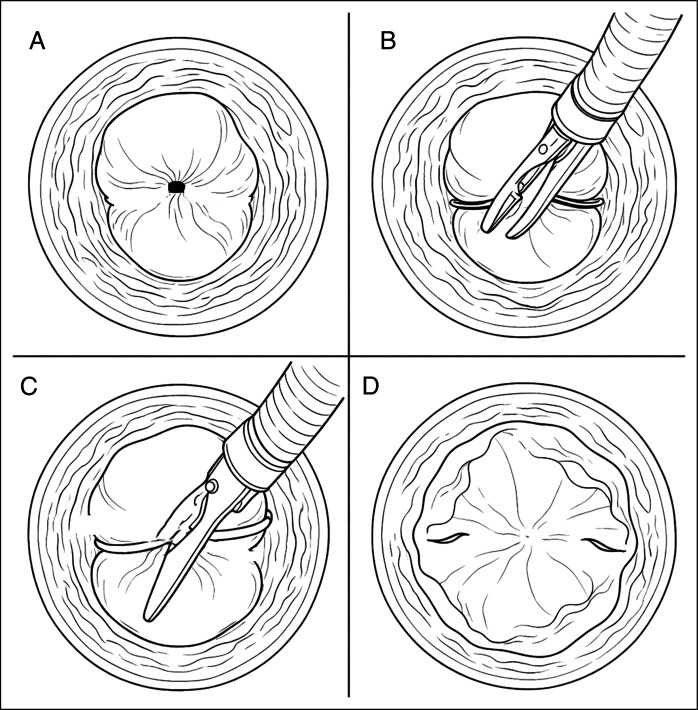
Simplified schematic diagram (A–D) of endoscopic release of an obstructing esophageal variceal band using endoscopic scissors. Panel (A) Top view cross-sectional esophagus constricted by elastic band causing near-complete luminal obstruction (button hole appearance; band not visible). Panel (B) Endoscope with closed scissors emerging from working channel approaching the band (side view; band visible). Panel (C) Open scissor blades positioned perpendicular across the elastic band without touching esophageal wall (side view). Panel (D) Band transected with restored lumen and decompressed varix (top view).

In our case, conservative management failed to relieve the obstruction after 48 hours, as serial imaging confirmed persistent dysphagia and no passage of contrast. For this reason, endoscopic scissors were used to release the band, followed by balloon dilation without stent placement (Figures 7 and 8). This restored the luminal patency and resolved the dysphagia. This outcome emphasizes that when the obstruction persists beyond the early period, timely endoscopic intervention may be necessary to avoid prolonged hospitalization and prevent complications associated with nutritional and esophageal mucosal vascular compromise.

Our case highlights the complexities in managing acute esophageal obstruction following EVBL. Given the persistence of our patient's symptoms, endoscopic intervention was deemed the most appropriate treatment, as conservative management alone proved insufficient.

## DISCLOSURES

Author contributions: O. Osagiede, M. Lopez Quinteros, MS Zayat, JC Petrov: Conception and design, collection, and interpretation of the clinical data, and drafting of the article. FJ Lukens, MS Wagh, JG Hashash, TA Woodward: Critical review of the article. O. Osagiede is the article guarantor.

Financial disclosure: We do not have any financial or non-financial potential conflicts of interest.

Informed consent was obtained for this case report.

## ABBREVIATIONS:

EGD: esophagogastroduodenoscopy
EVBL: endoscopic variceal band ligation
